# Hexahydrocannabinol (HHC) and Δ^9^-tetrahydrocannabinol (Δ^9^-THC) driven activation of cannabinoid receptor 1 results in biased intracellular signaling

**DOI:** 10.1038/s41598-024-58845-7

**Published:** 2024-04-22

**Authors:** Oleh Durydivka, Petr Palivec, Matej Gazdarica, Ken Mackie, Jaroslav Blahos, Martin Kuchar

**Affiliations:** 1 Institute of Molecular Genetics of the Czech Academy of Sciences, Videnska 1083, 142 20 Prague 4, Czech Republic; 2 Forensic Laboratory of Biologically Active Substances, Department of Chemistry of Natural Compounds, University of Chemistry and Technology Prague, Technicka 3, Prague, Czech Republic; 3 Department of Psychological and Brain Sciences, Gill Center for Molecular Bioscience, Indiana University, 1101 E. 10th St., Bloomington, IN 47405 USA; 4 Psychedelic Research Center, National Institute of Mental Health, Topolová 748, Klecany, Czech Republic

**Keywords:** Drug regulation, Public health, Biochemistry, Cell biology, Chemical biology, Drug discovery, Molecular biology, Chemistry

## Abstract

The *Cannabis sativa* plant has been used for centuries as a recreational drug and more recently in the treatment of patients with neurological or psychiatric disorders. In many instances, treatment goals include relief from posttraumatic disorders, anxiety, or to support treatment of chronic pain. Ligands acting on cannabinoid receptor 1 (CB1R) are also potential targets for the treatment of other health conditions. Using an evidence-based approach, pharmacological investigation of CB1R agonists is timely, with the aim to provide chronically ill patients relief using well-defined and characterized compounds from cannabis. Hexahydrocannabinol (HHC), currently available over the counter in many countries to adults and even children, is of great interests to policy makers, legal administrators, and healthcare regulators, as well as pharmacologists. Herein, we studied the pharmacodynamics of HHC epimers, which activate CB1R. We compared their key CB1R-mediated signaling pathway activities and compared them to the pathways activated by Δ^9^-tetrahydrocannabinol (Δ^9^-THC). We provide evidence that activation of CB1R by HHC ligands is only broadly comparable to those mediated by Δ^9^-THC, and that both HHC epimers have unique properties. Together with the greater chemical stability of HHC compared to Δ^9^-THC, these molecules have a potential to become a part of modern medicine.

## Introduction

The *Cannabis sativa* plant has been cultivated for recreational and medical use for centuries^1^. Various psychotropic and therapeutic effects of cannabis have been attributed to the major constituents of the plant, Δ^9^-tetrahydrocannabinol (Δ^9^-THC) and cannabidiol (CBD), and these compounds have been extensively studied for medical applications. Cannabis plant extracts include many compounds in addition to Δ^9^-THC and CBD. Over 400 different compounds have been isolated from the plant, including ligands of cannabinoid receptors, terpenes, alkaloids, and flavonoids^2,3^. A growing interest in these compounds has resulted in a systematic exploration of the therapeutic potential of other cannabis or cannabinoid-derived compounds. These compounds often have unique chemical, pharmacodynamic, and pharmacokinetic properties, differing from those of Δ^9^-THC or CBD, which may lead to novel therapeutic uses. Current discussion about the effects and safety of hexahydrocannabinol (HHC) use mandates an examination of its pharmacodynamic properties, including its effect on cannabinoid receptor 1 (CB1R), as there are only limited data concerning its activity, potency, toxicity, and safety^4,5^.

Δ^9^-THC-related cannabinoids share the Δ^9^-THC’s overall structure but differ in the position of a double bond, number and/or orientation of methyl groups, degree of hydrogenation, and length of the side chain. Importantly, these modifications affect their stability and pharmacological characteristics. One “minor” cannabinoid, HHC, is found in trace amounts in the cannabis plant^6^. This cannabinoid can be easily produced from CBD, initially undergoing acid cyclization to form Δ^9^-THC, which is subsequently hydrogenated to produce HHC. Due to its straightforward preparation and general availability of CBD, HHC is being manufactured at large scales and widely abused as a novel cannabinoid^7^.

**Figure 1:**
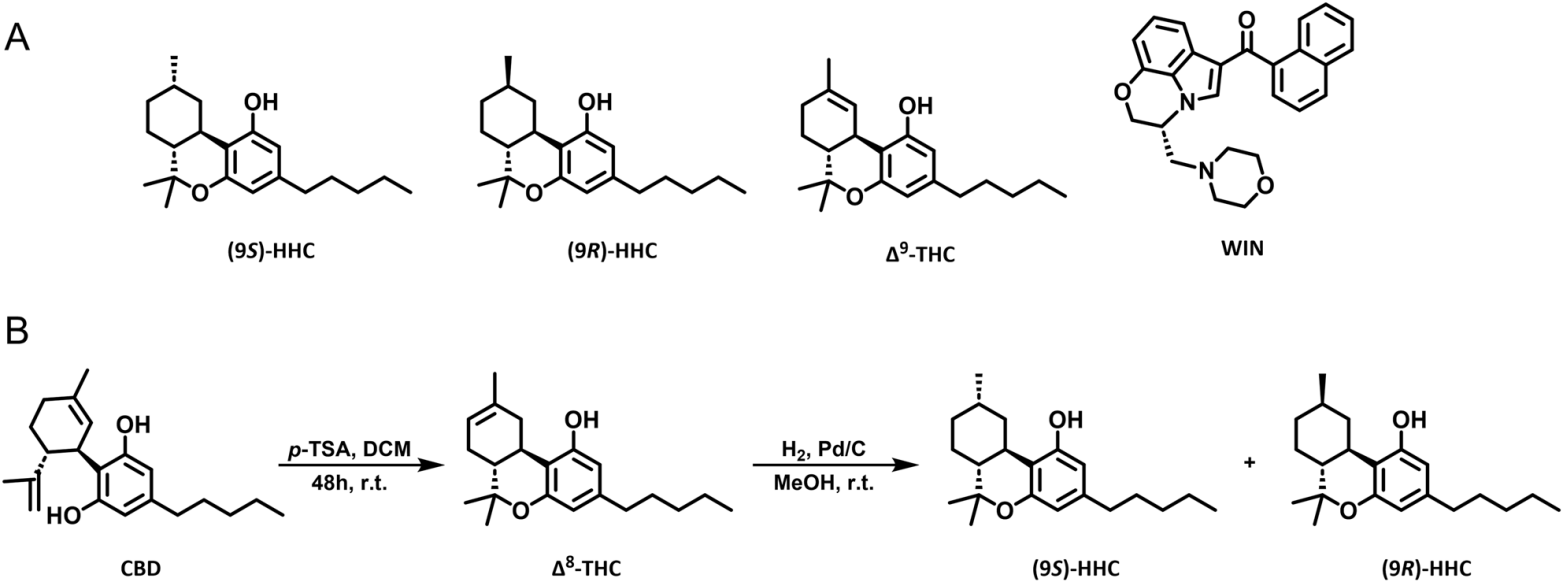
The structures of the tested cannabinoids and the HHC synthesis scheme. (**A**) The structures of (9*S*)-HHC, (9*R*)-HHC, Δ^9^-THC, and WIN. (**B**) The synthesis of HHC from CBD, schematically via transformation of CBD into Δ^8^-THC and further reduction to obtain HHC epimers.

Synthesis of HHC produces two epimers: (9*S*)-HHC and (9*R*)-HHC, which differ in the orientation of the methyl group at atom 9 (Fig. 1A). The (9*R*)-HHC epimer has superior affinity to cannabinoid receptor 1 over (9*S*)-HHC^8^. Early reports comparing HHC and its epimers were compromised by low purity of the compounds^9,10^. A recent study that used purified HHC epimers showed that the effect of (9*R*)-HHC on mouse behavior is close to that of Δ^9^-THC, while (9*S*)-HHC lacks Δ^9^-THC-like effects^11^.

Cannabinoids activate the cannabinoid receptor family consisting of cannabinoid receptor 1 and cannabinoid receptor 2, peroxisome proliferator-activated receptors (PPAR) α and γ, transient receptor potential cation channel subfamily V member 1 (TRPV1), and orphan receptors GPR55 and GPR18^12–16^, among others. However, the key target of Δ^9^-THC in the brain is CB1R, a G protein-coupled receptor that is widely expressed in the brain, especially in the hypothalamus, hippocampus, nucleus accumbens, prefrontal cortex, cerebellum, and the emetic centers in the brainstem^17^. Neuron-wise, CB1R is located presynaptically, where it may inhibit excitatory or inhibitory synaptic transmission^18^.

CB1R activation principally results in the activation of G_i/o_ proteins, decreasing levels of cyclic adenosine triphosphate (cAMP) in the cell and regulating several other signaling pathways. The activity of the receptor is controlled by G protein-coupled receptor kinase 3 (GRK3)-dependent phosphorylation and subsequent binding of β-arrestin; this inhibition of the receptor’s ability to elicit a response is known as desensitization. Further, β-arrestin initiates internalization of the receptor and, at the same time, facilitates activation of signaling pathways such as ERK1/2 or JNK3^19,20^.

Distinct outcomes of GPCR signaling that arise from different compounds activating unique networks of signaling pathways result in signaling bias. In signaling bias, each ligand preferentially activates a suite of particular signaling pathways. Known cannabinoid ligands can preferentially activate subsets of G protein- or β-arrestin-dependent signaling and have different receptor internalization efficacies when the receptors are expressed in heterologous systems^21,22^.

In the present study, we aimed to synthesize and purify (9*S*)-HHC and (9*R*)-HHC in larger amounts than previously accomplished, separate the epimers, and clarify their proportions in each procedure, thus allowing us to thoroughly explore the pharmacodynamics of the compounds at CB1R.

## Methods

### CB1R ligands

WIN 55,212-2 mesylate (WIN) was obtained from Tocris R&D (USA). Δ^9^-tetrahydrocannabinol (Δ^9^-THC) was synthesized as described previously^23^. Hexahydrocannabinol (HHC) was synthesized as described below.

### Synthesis and purification of HHC

Commercially-available CBD isolate (CBDepot, Czech Republic), *p*-toluenesulphonic acid (P-Lab, Czech Republic), and 5% palladium on activated charcoal (Merck KGaA, Germany) were used for the reaction. Solvents were purchased from a local distributor (Lach-Ner, Czech Republic) and were used without further purification. Solvents were evaporated using a vacuum rotary evaporator. Argon (5N) was used as an inert gas, and hydrogen (3.5N) was used for the reduction. Polar silica 40–63 µm (Merck KGaA, Germany) was used for ∆^8^-THC purification. The Aldrich^®^ Kugelrohr™ short-path distillation apparatus (Merck KGaA, Germany) was used for HHC vacuum distillation. HHC epimers were separated using COMBIFLASH RF200 UV/VIS (Teledyne ISCO, United States) and RediSep Gold^®^ Silica Gel Disposable Flash Columns (Teledyne ISCO, United States). HPLC/UV spectra were measured using LC/MS Agilent Technologies, 1290 Infinity DAD. The ratios of HHC epimers were determined based on signal characteristics in ^1^H NMR spectra (δ 3.03 ppm for (9*R*)-HHC and δ 2.87 for (9*S*)-HHC).

The scale of the reaction ranged from 10 g of CBD up to 1 kg. CBD was dissolved in DCM to achieve a concentration of 50 g/L. For every gram of CBD, 0.5 g of *p*-toluenesulphonic was added. The mixture was flushed with argon and stirred for 48 h at room temperature. The reaction mixture was filtered through the silica column using 3 g of silica for every gram of CBD. The silica was washed with DCM until no more product was eluted. The solution of ∆^8^-THC in DCM was concentrated to 1/10 of its original volume. An equal amount of MeOH was added diluting the solution approximately two times and the solution was evaporated once again to half of its volume. This procedure was repeated until no DCM signal (δ 5.30 ppm) was present on ^1^H NMR. The resulting mixture of ∆^8^-THC and MeOH was used for the reduction without further purification.

The corresponding conditions are listed in the Table 1. Palladium on activated charcoal was added to the solution of ∆^8^-THC in MeOH. The reaction vessel was flushed with argon and then the argon was replaced by hydrogen. The mixture was stirred, and the pressure of hydrogen was maintained at around 1 atm. The mixture was filtered through celite and the celite was washed with MeOH until no more product was eluted. The MeOH was evaporated and the crude HHC was vacuum distilled using Kugelrohr™ (220 °C, 0.4 torr). A mixture of epimers (9*R*/*S*)-HHC (HPLC/UV purity 96%) was produced by this procedure. Samples of pure (9*R*)-HHC and (9*S*)-HHC were obtained from a 3:2 mixture (entry 2) using FLASH chromatography (hexane: *t*-BuOMe, 1–2%).

**Table 1:** The synthesis of (9R)-HHC and (9S)-HHC at different scales.

Entry	Synthesis scale (∆^8^-THC mass), g	Mass of 5% Pd/C per 1 g of ∆^8^-THC, mg	∆^8^-THC concentration, g/L	Duration of the reduction, days	9*R*:9*S* molar ratio
1	10	50	200	2	3:1
2	10	20	50	2	3:2
3	250	50	200	5	3:1
4	1000	50	250	14	3:1

### NMR characterization of HHC epimers

The NMR spectra were measured with Agilent 400 MR DDR2 (Agilent Technologies Inc., United States) using CDCl_3_ (Merck KGaA, Germany) as a solvent and referenced on residual CDCl_3_ signal (^1^H δ 7.26 ppm). The spectra of corresponding epimers were identical to NMR spectra published by Russo et al.^11^.

#### (9S)-HHC

^1^H NMR (400 MHz, CDCl_3_) δ 6.25 (d, J = 1.6 Hz, 1H), 6.07 (d, J = 1.6 Hz, 1H), 4.70 (s, 1H), 2.91–2.85 (m, 1H), 2.71–2.64 (m, 1H), 2.47–2.37 (m, 2H), 2.15–2.07 (m, 1H), 1.69–1.61 (m, 3H), 1.56 (p, J = 7.6 Hz, 2H), 1.51–1.44 (m, 1H), 1.36 (s, 3H), 1.35–1.27 (m, 6H), 1.13 (d, J = 7.3 Hz, 3H), 1.09 (s, 3H), 0.88 (t, J = 7.0 Hz, 3H).

#### (9R)-HHC

^1^H NMR (400 MHz, CDCl_3_) δ 6.25 (d, J = 1.7 Hz, 1H), 6.08 (d, J = 1.6 Hz, 1H), 4.69 (s, 1H), 3.06–3.00 (m, 1H), 2.49–2.38 (m, 3H), 1.88–1.81 (m, 2H), 1.68–1.59 (m, 1H), 1.58–1.50 (m, 2H), 1.49–1.38 (m, 1H), 1.37 (s, 3H), 1.35–1.24 (m, 4H), 1.17–1.02 (m, 2H), 1.07 (s, 3H), 0.94 (d, J = 6.6 Hz, 3H), 0.88 (t, J = 7.0 Hz, 3H), 0.83–0.74 (m, 1H).

### Cell culture and transfection

Human Embryonic Kidney 293 (HEK293) cells (ATCC, USA, CRL-1573) were cultured in high glucose Dulbecco’s Modified Eagle’s Medium (DMEM) (Sigma) supplemented with 10% fetal bovine serum (Gibco) at 37 °C, 5% CO_2_ in the air, and 95% humidity. The cells were plated in 96-well plates (Greiner BioOne, UK) at 50,000 cells per well and transfected with 150 ng of DNA per well using Lipofectamine 2000 (Invitrogen) according to the manufacturer's instructions. The transfected cells were tested 24 h after transfection.

### Bioluminescence resonance energy transfer assay

Bioluminescence resonance energy transfer (BRET) assay was used to measure CB1R-induced G protein dissociation and β-arrestin interaction with CB1R, as described previously^24,25^. To evaluate G protein dissociation, we transfected the cells with G_αi1_-Rluc8 or G_αoA_-Rluc8, G_β2_-Flag, G_γ2_-EYFP, and SNAP-CB1R plasmids in a mass ratio of 1:1:1:2. To measure β-arrestin2 interaction with CB1R, we transfected the cells with β-arrestin2-Rluc and CB1R-EYFP plasmids in a mass ratio of 1:2. To study GRK3-CB1R interaction, the cells were transiently transfected with GRK3-Rluc8 and CB1R-EYFP plasmids (1:2 ratio). Before the measurements, the transfected cells were washed with phosphate-buffered saline (137 mM NaCl, 2.7 mM KCl, 8 mM Na_2_HPO_4_, 1.8 mM KH_2_PO_4_) and incubated in Tyrode’s solution (137 mM NaCl, 0.9 mM KCl, 1 mM MgCl_2_, 1 mM CaCl_2_, 11.9 mM NaHCO_3_, 3.6 mM NaH_2_PO_4_, 5.5 mM D-glucose, 25 mM HEPES, pH 7.4) at 37 °C for at least 30 min. Next, we added coelenterazine h (NanoLight) at a final concentration of 5 µM to the cells, followed by the addition of increasing concentrations of compounds (9*S*)-HHC, (9*R*)-HHC, Δ^9^-THC, WIN, or their vehicles. BRET donor and acceptor emission was measured 12 min after the addition of the compounds using Mithras LB940 plate reader (Berthold Biotechnologies, Germany). The BRET ratio was obtained by dividing the acceptor emission (540 ± 20 nm) by the donor emission (480 ± 10 nm). After subtracting the BRET ratio of the vehicle addition from the BRET ratio of the compounds, we obtained deltaBRET (ΔBRET). Data analysis was performed using GraphPad Prism 9.3.1 for Windows (GraphPad Software, USA). The concentration–response curves were fitted using a non-linear regression function.

### CB1R internalization assay

Cell surface receptor internalization was studied using the Homogenous Time-Resolved FRET (HTRF) technology as described previously^26^. First, HEK293 cells were seeded on a 96-well plate (Merck, Germany) and transiently transfected with SNAP-tagged CB1R plasmid together with empty vector pRK6 (1:2 DNA mass ratio) using Lipofectamine™ 2000 (Thermo Fisher Scientific) according to the manufacturer's protocol. Twenty-four hours post-transfection, the cell culture medium was removed, and the cells were labeled with 100 nM SNAPLumi4-Tb (PerkinElmer—CisBio, France), diluted in Tag-lite labeling medium (PerkinElmer—CisBio, France) and incubated for 1 h at 37 °C, 5% CO_2_. Subsequently, labeled cells were washed four times with Tag-Lite buffer solution. The receptor internalization experiment was performed by adding Tag-lite buffer containing 24 μM fluorescein (Merck, Germany) and corresponding CB1R agonist or vehicle (dimethyl sulfoxide, or in the case of Δ^9^-THC, ethanol). HTRF signal was recorded over 60 min at 37 °C using the Mithras LB 940 microplate reader (Berthold Technologies, Germany) equipped with the HTRF module and relevant filters. The donor fluorophore (terbium cryptate) was excited at 340 ± 26 nm and emission was measured at 520 ± 10 nm. The acceptor (fluorescein) emission was measured at 620 ± 10 nm. The HTRF ratio was calculated as the donor emission divided by the acceptor emission multiplied by 10,000.

### Extracellular signal-regulated kinases 1/2 phosphorylation assay

Phosphorylation levels of endogenous extracellular signal-regulated kinases 1/2 (ERK1/2) were detected using the Phospho-ERK1/2 (Thr202/Tyr204) kit (Cisbio Bioassays, France). The transfected cells plated in 96-well plates (Greiner BioOne, UK) were serum-starved for 16 h prior to the experiment in serum-free DMEM media. Afterwards, the cells were stimulated for the indicated times by CB1R ligand diluted in serum-free DMEM and then lysed in 50 μl of supplemented lysis buffer. After homogenization, 16 μl of the cell lysate was transferred from the 96-well plate to a 384-well black plate (Greiner BioOne, UK) and incubated with 4 μl of detection buffer containing anti-ERK1/2-Eu^3+^cryptate and anti-Phospho-ERK1/2-d2 for at least 4 h in dark. The fluorescence emissions at 665 nm and 620 nm were read on HTRF® compatible Mithras LB 940 microplate reader (Berthold Technologies, Germany). Data are presented as the ratio of 665 nm emission and 620 nm emission multiplied by 10,000.

## Results

### Synthesis of (9S)-HHC and (9R)-HHC

We produced the HHC epimers by employing the ∆^8^-THC reduction reaction, described previously by Russo and colleagues (Fig. 1B)^11^. They found that using ∆^8^-THC for the reaction provides predominantly (9*R*)-HHC in a 3:1 ratio. They also claimed that using ∆^9^-THC as a precursor leads to an excess of (9*S*)-HHC in a 2:1 ratio^11^. Because we used ∆^8^-THC in the reduction reaction, we were able to confirm that, when a high concentration of ∆^8^-THC and a relatively high amount of palladium on carbon is used, the reaction indeed predominantly produces (9*R*)-HHC at a ratio of approximately 3:1 (Table 1, entry 1). However, when only a small amount of palladium on carbon and a low concentration was used, (9*R*)-HHC was produced in a lower ratio of 3:2 (entry 2). This reaction was also carried out in separate preparations at larger scales of 250 g (entry 3) and 1 kg (entry 4). For the scale-up reaction, a high concentration of ∆^8^-THC and high amount of palladium on carbon was used, yielding predominantly (9*R*)-HHC in a 3:1 ratio. Achieving complete hydrogenation at large scales took significantly longer than at a small scale. Yields of HHC were not calculated as the hydrogenation reaction is quantitative and yields are mostly dependent on the scale of the reaction due to losses during distillation.

### G protein activation induced by CB1R stimulation

**Figure 2:**
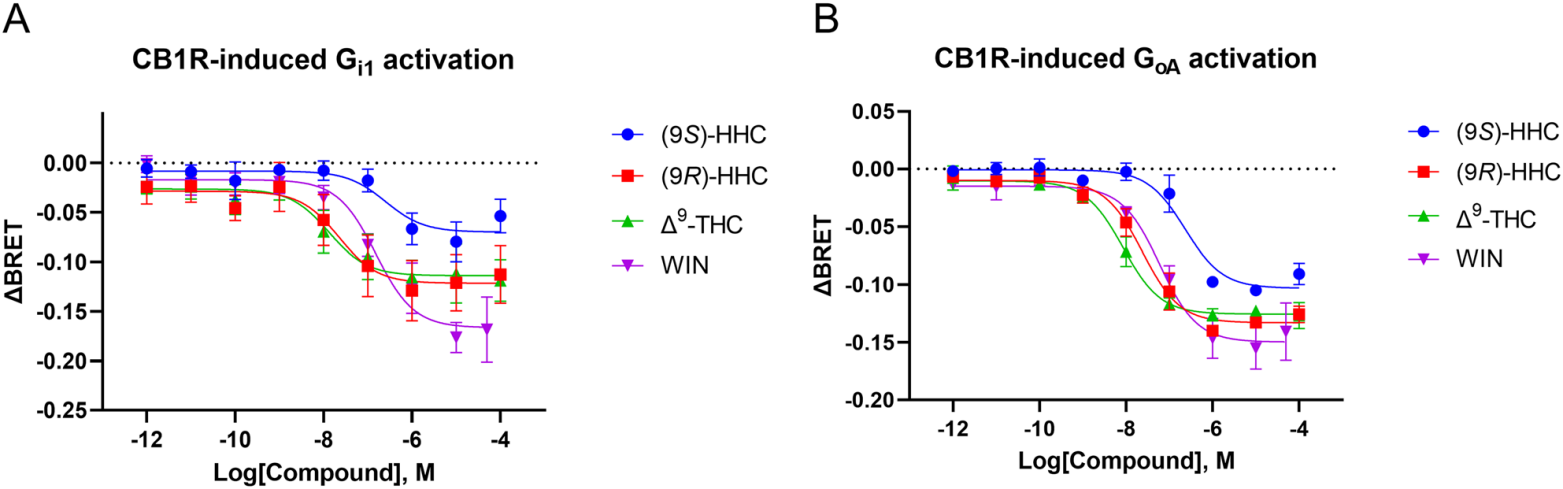
CB1R-driven G protein activation induced by the tested cannabinoids. HEK293 cells were transiently transfected with G_αi1_-Rluc8 or G_αoA_-Rluc8, G_β2_-Flag, G_γ2_-VENUS, and SNAP-CB1R. The cells were stimulated with the indicated concentrations of (9*S*)-HHC, (9*R*)-HHC, Δ^9^-THC, WIN, or their vehicles. BRET donor and acceptor emission was measured 12 min after receptor stimulation. (**A**) Concentration–response relationship of G_αi1_ subunit dissociation from the G protein complex after CB1R stimulation. (**B**) Concentration–response relationship of G_αoA_ subunit dissociation from the G protein complex after CB1R stimulation. The data are presented as means ± SEM from three independent experiments. The data analysis is disclosed in Supplementary Tables 1 and 2.

To test whether HHC induces signaling via CB1R, we first measured the G protein activation in the transfected cells. We tested the effect of the studied cannabinoids (9*S*)-HHC, (9*R*)-HHC, Δ^9^-THC, and WIN on G protein activation by employing a BRET-based assay that monitors the dissociation of G_α_ and G_βγ_ subunits of the G_i/o_ protein upon its activation by CB1R. We tested G_i1_ and G_oA_ activation mediated by CB1R stimulation with increasing concentrations of (9*S*)-HHC, (9*R*)-HHC, Δ^9^-THC, and WIN. In all cases, agonist engagement of the receptor was followed by a prompt decrease in the BRET ratio, reflecting activation of the G proteins (Fig. 2).

In the G_i1_ and G_oA_ activation assays, (9*S*)-HHC had a potency and efficacy lower than (9*R*)-HHC (Fig. 2 and Supplementary Tables 1 and 2). The potency and efficacy of (9*R*)-HHC were similar to those of Δ^9^-THC. Overall, the results demonstrate that the effect of (*9R*)-HHC epimer on the G_i_ and G_o_ signaling pathways is similar to that of Δ^9^-THC, while (9*S*)-HHC induces lower levels of the G protein activation.

### GRK3 and β-arrestin2 interactions with the activated CB1R

**Figure 3:**
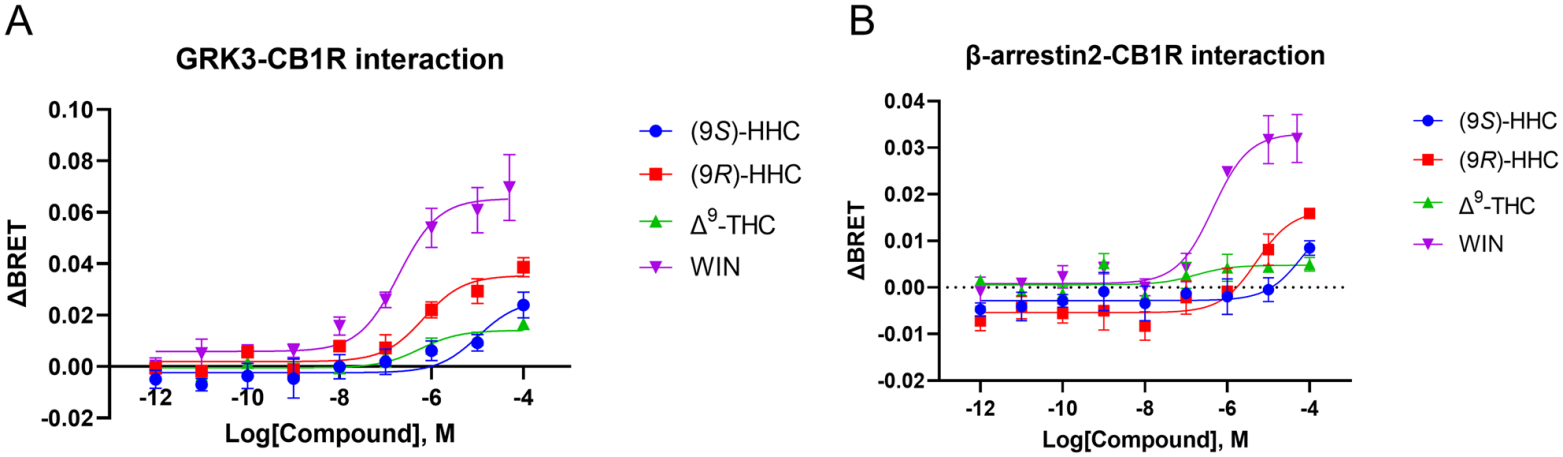
GRK3-CB1R and β-arrestin2-CB1R interactions elicited by the tested cannabinoids. HEK293 cells were transiently transfected with CB1R-EYFP and β-arrestin2-Rluc or GRK3-Rluc8 (1:2 ratio). The cells were stimulated with increasing concentrations of compounds (9*S*)-HHC, (9*R*)-HHC, Δ^9^-THC, WIN, or their vehicles. BRET donor and acceptor emission was measured 12 min after the addition of the compounds. (**A**) Concentration–response relationship of GRK3-CB1R association mediated by CB1R stimulation. (**B**) Concentration–response relationship of β-arrestin2 recruitment to CB1R after CB1R stimulation. The data are presented as means ± SEM from three independent experiments. The data analysis is disclosed in Supplementary Tables 3 and 4.

We next studied the recruitment of GRK3 and β-arrestin2 to CB1R, as stimulated by the tested cannabinoids. The employed BRET-based interaction assays monitor the association of GRK3 and CB1R following receptor phosphorylation and the interaction of β-arrestin2 with the phosphorylated CB1R. Agonist activation of the receptor increased the BRET ratio, reflecting increased GRK3-CB1R and β-arrestin2-CB1R interactions (Fig. 3).

WIN application in these assays elicited the strongest responses and also showed the highest potency and efficacy (Fig. 3 and Supplementary Tables 3 and 4). On the other hand, the interactions induced by Δ^9^-THC were negligible. The potency of (9*R*)-HHC was higher than that of (9*S*)-HHC, but the curve fitting demonstrated that these epimers have similar efficacies. Overall, the results indicate that the (9*R*)-HHC epimer stimulates GRK3-CB1R and β-arrestin2-CB1R interactions more effectively than Δ^9^-THC or the (9*S*)-HHC epimer.

### Internalization of activated CB1R

β-arrestin interaction with the desensitized receptor initiates receptor internalization and activates specific signaling cascades. We used the HTRF-based approach to monitor the kinetics of receptor internalization upon activation by the tested cannabinoids. In this approach, internalization results in increased emission of the terbium cryptate fluorophore that is covalently attached to the receptor.

**Figure 4:**
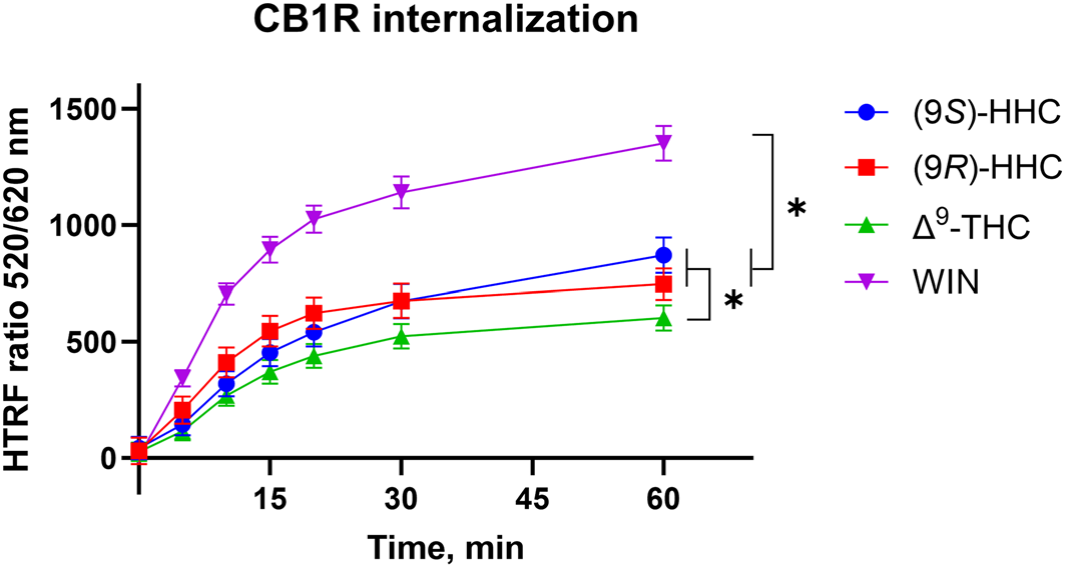
Internalization of CB1R induced by the tested cannabinoids. Internalization was elicited by the application of 10 µM of (9*S*)-HHC, (9*R*)-HHC, Δ^9^-THC, and WIN. HEK293 cells were transiently transfected with the plasmids coding SNAP-CB1R or mock plasmid pRK6 (1:2 DNA mass ratio). Data represent net receptor internalization by each drug treatment (i.e. receptor internalization by the indicated drug minus receptor internalization by vehicle). The data are presented as means ± SEM of three independent experiments performed in 3 technical replicates. The statistical analysis is disclosed in Supplementary Table 5. *, p < 0.05 by ANOVA.

Application of the cannabinoids initiated prompt and massive internalization of CB1R, but the extent of internalization varied. WIN had the highest effect on CB1R internalization rate (Fig. 4). The HHC epimers and Δ^9^-THC had comparable effects on CB1R internalization, which were about half of the WIN effect.

### ERK1/2 phosphorylation induced by CB1R stimulation

G proteins and β-arrestin both contribute to the activity of the ERK1/2 signaling cascade. We determined the extent of ERK1/2 activity driven by the tested cannabinoids by measuring its phosphorylation in the HTRF-based sandwich ELISA. In this assay, ERK1/2 phosphorylation is detected as an increase in the HTRF ratio.

**Figure 5:**
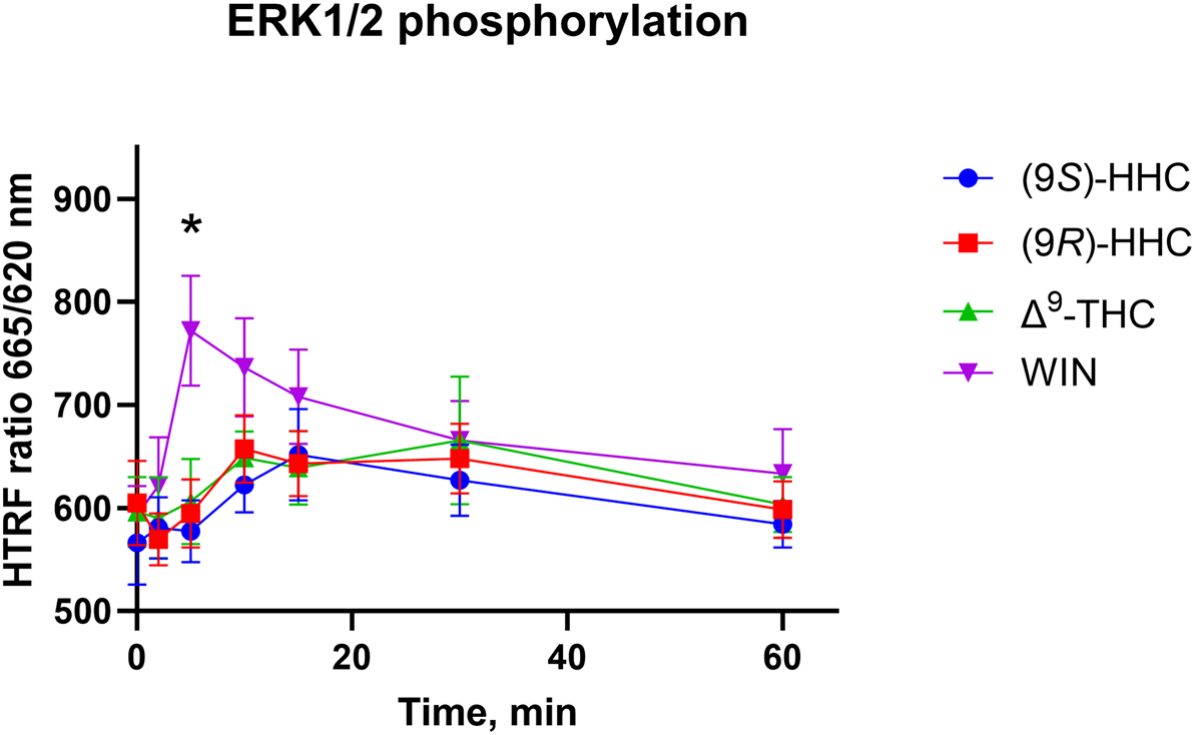
ERK1/2 phosphorylation elicited by the cannabinoids. HEK293 cells were transiently transfected with CB1R and empty vector (1:2 ratio). 24 h after transfection, cells were stimulated by 10 µM of (9*S*)-HHC, (9*R*)-HHC, Δ^9^-THC or WIN, and kinetics of ERK1/2 phosphorylation were measured at the indicated times. The data are presented as means ± SEM of three independent experiments performed in 3 technical replicates. The statistical analysis is disclosed in Supplementary Table 6. *, p < 0.05 (9*S*)-HHC vs. WIN by ANOVA.

WIN activation of CB1R led to a rapid but transient increase in ERK1/2 phosphorylation that peaked at 5 min after agonist stimulation and then progressively diminished (Fig. 5). Application of (9*R*)-HHC, (9*S*)-HHC, and Δ^9^-THC induced lower levels of ERK1/2 phosphorylation, peaking at 10 min.

## Discussion

CB1R is the principal receptor of the central nervous system endocannabinoid system (ECS)^27^. CB1R is expressed in all brain regions, including those important for processing anxiety, fear, stress, and cognitive functions. CB1R is abundant in the basal ganglia, hippocampus, cerebellum, prefrontal cortex, and amygdala^28^. The neuronal ECS, with its central receptor, CB1R, is important for synaptic plasticity, strength, and maintenance. In addition to neurons, CB1R is also expressed in the central nervous system in astrocytes, microglia, and oligodendrocytes, where it modulates synaptic transmission, glucose metabolism, and immunomodulator production^18,29^. Furthermore, CB1R is also present in the peripheral nervous system, as well as in skeletal muscle, bone, skin, eyes, adipose tissue, and the reproductive system^30^. Subcellularly, CB1R is typically, but not exclusively, located presynaptically in many glutamatergic, GABAergic, cholinergic, serotonergic, and noradrenergic neurons. Endocannabinoids are synthesized on demand on the postsynaptic side and suppress neurotransmitter release via activation of presynaptic CB1R^31–33^. CB1R is primarily directed to cell surface; however, an important discrete pool of CB1Rs is in the outer mitochondrial membrane^34^.

ECS is involved in appetite stimulation, energy balance regulation, learning and memory, pain processing, neurogenesis and neuroprotection, immune responses, and many other physiological regulations including neurohumoral system homeostasis. CB1R also plays an important role in pathological conditions including schizophrenia, multiple sclerosis, anxiety, depression, epilepsy, Parkinson's disease, Huntington's disease, Alzheimer's disease, addiction, stroke, inflammation, glaucoma, cancer, as well as musculoskeletal and liver disorders^16,35^.

The *Cannabis sativa* plant produces a vast repertoire of chemically and biologically interesting and diverse compounds. Over 400 compounds, about a quarter of which unique, have been detected in the plant. This remarkable mixture includes phytocannabinoids, terpenes and other compound classes^2,3^. Recent efforts to use a scientific approach to marijuana for medical purposes, namely in Canada, Israel, the USA, and the Czech Republic, have led to an approach in which two main substances, Δ^9^-THC and CBD, were evaluated by controlled trials in a broad cohort of patients with favorable outcomes. However, it is known that *Cannabis sativa* chemistry is not limited to only these two compounds, and many more structures must be taken into an account.

One historically-overlooked CB1R ligand, HHC, share a similar chemical structure with Δ^9^-THC and CBD. Herein we show that they activate CB1R in a unique way, most likely by favoring differential active conformational states of CB1R than those favored by Δ^9^-THC. Recent studies in mice have shown that HHC compounds are psychoactive, namely in the cannabinoid tetrad tests. Many results from behavioral analyses highlight generally overlapping, but not entirely parallel impacts, on the performance in the tests. The pharmacodynamic analyses presented here, together with subsequent pharmacokinetic studies may help us to understand these differences.

Various examples of ligands that exert divergent effects on CB1R signaling pathways have been described. Certain cannabinoids favor G protein-mediated signaling over the β-arrestin pathway, as in the case of novel compounds PNR-4-20 and PNR-4-02 that selectively activate the G_αi_ pathway, while eliciting significantly less β-arrestin2 recruitment^36^. On the other hand, the allosteric modulator ORG27569 induces CB1R conformation state that selectively activates the ERK1/2 cascade via β-arrestin1^37^. Distinct ligands induce and stabilize different conformations of a given GPCR. Consequently, these conformations could preferentially activate a particular signaling cascade over others, a phenomenon called “biased signaling”. Activation of a pathway resulting in desired therapeutic efficacy, together with a decrease of signaling pathways leading to undesired effects, typically psychoactivity or tolerance, may have profound consequences in drug discovery of molecules with potential medicinal uses, including those acting via CB1R. CB1R-mediated signaling is complex, and its outcome depends on the cellular environment, associated protein network, and ligand that activates the receptor in a particular way, or modulates its signaling in a unique way for each HHC enantiomer.

In neurons and other naïve cells, CB1R-interacting proteins also bias the signaling of the receptor, for example, SH3-containing GRB2-like protein 3-interacting protein 1 (SGIP1)^24,25,38,39^, Cannabinoid Receptor Interacting Protein 1a and 1b (CRIP1a/b)^40,41^, and G Protein-Coupled Receptor Associated Protein 1 (GASP1)^42,43^. The situation may become yet more complex with heterodimers of CB1R^44,45^. For example, CB1R was reported to form dimers with dopamine receptor 2. Activation of these heterodimeric receptors may activate the G_αs_ pathway leading to the increase of cAMP, thus generating the opposite effect as when CB1R is signaling alone^44^.

**Figure 6:**
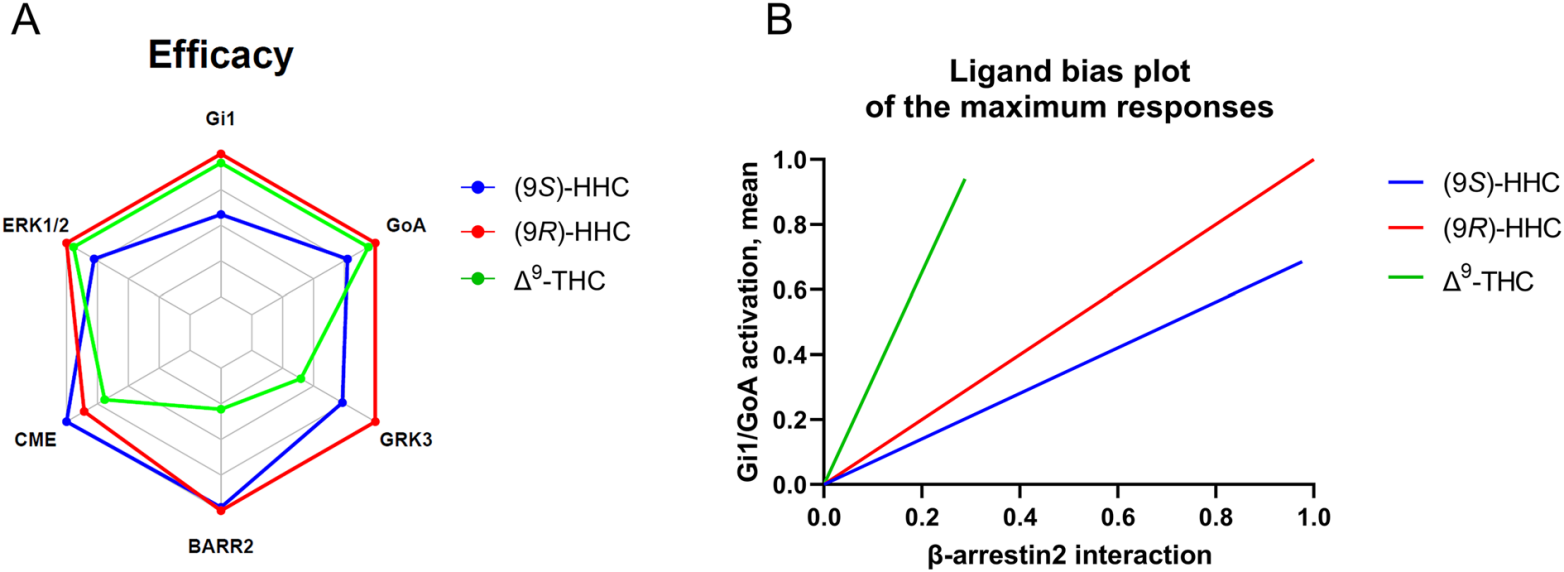
Pharmacological profiles of (9S)-HHC, (9R)-HHC, and Δ^9^-THC. (**A**) Calculated maximum response values or the time-course peaks were plotted on the axes of the radar plot. For clathrin-mediated internalization (CME), the 60 min time points were used; for ERK1/2 phosphorylation, the 10 min time points were used. (**B**) Calculated maximum response values of the tested cannabinoids were represented as fractions of WIN and normalized to (9*R*)-HHC. The normalized values for β-arrestin interaction were plotted on the x-axis, and the means of the normalized values for G_i1_/G_oA_ activation were plotted on the y-axis. The values represent only the maximum responses elicited by the ligands.

The urgent need for better pharmaceutical management in patients prompts investigations for novel therapeutic agents. The ECS is involved in a plethora of nervous system physiology and pathophysiology. However, implementing medical applications achieved by manipulating the ECS has been challenging, mainly due to the pleiotropic functions of the ECS. These include psychoactive and other undesired side effects of drugs acting on CB1R. Pharmacological approaches based on tinkering with the pleiotropic nature of CB1R signaling are one way to avoid undesired side effects. The biased CB1R-mediated signaling of the two HHC epimers, compared with each other and that of Δ^9^-THC (Fig. 6), together with the greater stability of HHC, represents an emerging prospective treatment via the ECS with possibly limited side effects.

### Supplementary Information

Supplementary Information.

## Data Availability

The datasets generated during and/or analysed during the current study are available from the corresponding authors on reasonable request.

## Abbreviations

HHC: Hexahydrocannabinol
(9*S*)-HHC: (9*S*)-6,6,9-Trimethyl-3-pentyl-6a,7,8,9,10,10a-hexahydro-6*H*-benzo[*c*]chromen-1-ol
(9*R*)-HHC: (9*R*)-6,6,9-Trimethyl-3-pentyl-6a,7,8,9,10,10a-hexahydro-6*H*-benzo[*c*]chromen-1-ol
MeOH: Methanol
Δ^8^-THC: Δ^8^-Tetrahydrocannabinol
Δ^9^-THC: Δ^9^-Tetrahydrocannabinol
BRET: Bioluminescence resonance energy transfer
cAMP: Cyclic adenosine triphosphate
CB1R: Cannabinoid receptor 1
CRIP1a/b: Cannabinoid receptor interacting protein 1a and 1b
DNA: Deoxyribonucleic acid
DCM: Dichloromethane
DMEM: Dulbecco’s modified Eagle’s medium
ECS: Endocannabinoid system
GASP1: G protein-coupled receptor associated protein 1
GRK3: G protein-coupled receptor kinase 3
HEK293: Human embryonic kidney 293
HTRF: Homogenous time-resolved FRET
HPLC: High-performance liquid chromatography
NMR: Nuclear magnetic resonance
PPAR: Peroxisome proliferator-activated receptor
SGIP1: SH3-containing GRB2-like protein 3-interacting protein 1
TRPV1: Transient receptor potential cation channel subfamily V member 1
EYFP: Enhanced yellow fluorescent protein
