# Supplementary material for: Hexahydrocannabinol (HHC) and Δ9-tetrahydrocannabinol (Δ9-THC) driven activation of cannabinoid receptor 1 results in biased intracellular signaling
Source: Sci Rep. 2024 Apr 22;14:9181. doi: 10.1038/s41598-024-58845-7 (PMC11035541; doi:10.1038/s41598-024-58845-7)
Supplement: Supplementary file 1 — Supplementary Information. [file 41598_2024_58845_MOESM1_ESM.pdf]

Hexahydrocannabinol (HHC) and Δ^{9}-tetrahydrocannabinol (Δ^{9}-THC) driven activation of cannabinoid receptor 1 results in biased intracellular signaling

Oleh Durydivka, Petr Palivec, Matej Gazdarica, Ken Mackie, Jaroslav Blahos & Martin Kuchar

Supplementary Information

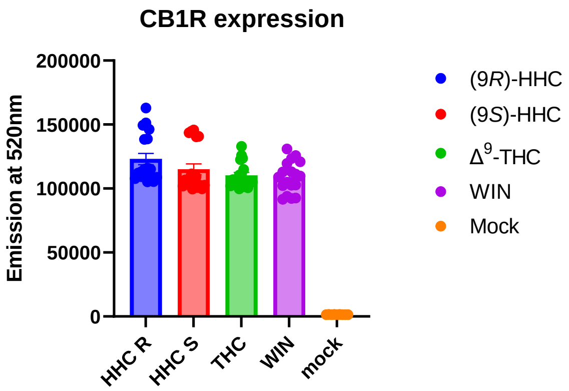

Supplementary Figure 1. Expression levels of SNAP-CB1R in transiently transfected HEK293 cells used in the receptor internalization assay (related to Fig. 4). Emission of SNAPLumi4-Tb-tagged CB1R-SNAP was measured at 520 ± 10 nm upon excitation at 340 ± 26 nm on Mithras LB 940 microplate reader.

Supplementary Table 1. Curve-fitting analysis of the G_{i1} activation data, presented in Figure 2. LogEC50 and EC50 represent ligand potency, and Top represents ligand efficacy.

**Compound**	**(9*S*)-HHC**	**(9*R*)-HHC**	**Δ⁹-THC**	**WIN**
**Best-fit values**				
**Bottom**	-0.008036	-0.02847	-0.02623	-0.01680
**Top**	-0.06988	-0.1214	-0.1137	-0.1664
**LogEC50**	-6.597	-7.650	-7.876	-6.818
**EC50**	2.532e-007	2.238e-008	1.330e-008	1.522e-007
**Span**	-0.06184	-0.09290	-0.08749	-0.1496
**95% CI (profile likelihood)**				
**Bottom**	-0.01980 to 0.003972	-0.05146 to -0.004468	-0.04467 to -0.007311	-0.03316 to -0.0002765
**Top**	-0.08842 to -0.05251	-0.1484 to -0.09620	-0.1339 to -0.09491	-0.1921 to -0.1434
**LogEC50**	-7.327 to -5.928	-8.599 to -6.712	-8.648 to -6.966	-7.336 to -6.229
**EC50**	4.705e-008 to 1.179e-006	2.515e-009 to 1.943e-007	2.248e-009 to 1.081e-007	4.612e-008 to 5.905e-007
**Goodness of Fit**				
**Degrees of Freedom**	24	24	24	24
**R squared**	0.6062	0.5710	0.6702	0.8489
**Sum of Squares**	0.01192	0.03509	0.02047	0.01982
**Sy.x**	0.02229	0.03824	0.02921	0.02874

Supplementary Table 2. Curve-fitting analysis of the G_{oA} activation data, presented in Figure 2. LogEC50 and EC50 represent ligand potency, and Top represents ligand efficacy.

**Compound**	**(9*S*)-HHC**	**(9*R*)-HHC**	**Δ⁹-THC**	**WIN**
**Best-fit values**				
**Bottom**	-0.0007050	-0.009940	-0.01025	-0.01486
**Top**	-0.1031	-0.1330	-0.1255	-0.1498
**LogEC50**	-6.633	-7.623	-8.069	-7.223
**EC50**	2.329e-007	2.381e-008	8.529e-009	5.986e-008
**Span**	-0.1024	-0.1230	-0.1153	-0.1349
**95% CI (profile likelihood)**				
**Bottom**	-0.008361 to 0.007019	-0.01821 to -0.001553	-0.01768 to -0.002737	-0.02691 to -0.002565
**Top**	-0.1148 to -0.09178	-0.1423 to -0.1238	-0.1329 to -0.1182	-0.1654 to -0.1345
**LogEC50**	-6.911 to -6.357	-7.884 to -7.357	-8.298 to -7.846	-7.611 to -6.872
**EC50**	1.228e-007 to 4.393e-007	1.307e-008 to 4.397e-008	5.036e-009 to 1.425e-008	2.447e-008 to 1.341e-007
**Goodness of Fit**				
**Degrees of Freedom**	24	24	24	24
**R squared**	0.9117	0.9496	0.9581	0.9015
**Sum of Squares**	0.004908	0.004340	0.003158	0.01042
**Sy.x**	0.01430	0.01345	0.01147	0.02084

Supplementary Table 3. Curve-fitting analysis of the GRK3-CB1R interaction data, presented in Figure 3. LogEC50 and EC50 represent ligand potency, and Top represents ligand efficacy.

Compound	(9*S*)-HHC	(9*R*)-HHC	Δ^9-THC	WIN
**Best-fit values**				
**Bottom**	-0.002394	0.001923	-0.0004729	0.005882
**Top**	0.02599	0.03543	0.01409	0.06538
**LogEC50**	-4.983	-6.172	-6.250	-6.712
**EC50**	1.041e-005	6.731e-007	5.624e-007	1.940e-007
**Span**	0.02838	0.03351	0.01456	0.05950
**95% CI (profile likelihood)**				
**Bottom**	-0.006911 to 0.001333	-0.001247 to 0.005034	-0.003035 to 0.001880	1.282e-005 to 0.01166
**Top**	0.01168 to 0.5234	0.02976 to 0.04153	0.009846 to 0.01960	0.05691 to 0.07450
**LogEC50**	-7.057 to -2.711	-6.690 to -5.700	-7.324 to -5.146	-7.163 to -6.245
**EC50**	8.780e-008 to 0.001943	2.044e-007 to 1.997e-006	4.737e-008 to 7.138e-006	6.872e-008 to 5.690e-007
**Goodness of Fit**				
**Degrees of Freedom**	24	24	24	24
**R squared**	0.5735	0.8513	0.6729	0.8692
**Sum of Squares**	0.001474	0.0008500	0.0004568	0.002604
**Sy.x**	0.007837	0.005951	0.004363	0.01042

Supplementary Table 4. Curve-fitting analysis of the β-arrestin2-CB1R interaction data, presented in Figure 3. LogEC50 and EC50 represent ligand potency, and Top represents ligand efficacy.

Compound	(9*S*)-HHC	(9*R*)-HHC	Δ^9-THC	WIN
**Best-fit values**				
**Bottom**	-0.002803	-0.005400	0.0005880	0.0008429
**Top**	0.01618	0.01657	0.004772	0.03307
**LogEC50**	-4.165	-5.284	-6.765	-6.344
**EC50**	6.846e-005	5.200e-006	1.717e-007	4.526e-007
**Span**	0.01899	0.02197	0.004184	0.03223
**95% CI (profile likelihood)**				
**Bottom**	-0.004790 to -0.0008480	-0.007917 to -0.002964	-0.001292 to 0.002402	-0.001783 to 0.003441
**Top**	0.004019 to ND	0.009883 to 0.02542	0.002288 to 0.009830	0.02875 to 0.03762
**LogEC50**	-5.513 to ND	-6.082 to -4.612	-10.35 to -3.970	-6.687 to -6.013
**EC50**	3.070e-006 to ND	8.281e-007 to 2.444e-005	4.417e-011 to 0.0001070	2.056e-007 to 9.701e-007
**Goodness of Fit**				
**Degrees of Freedom**	24	24	24	24
**R squared**	0.4298	0.7050	0.2390	0.8877
**Sum of Squares**	0.0004427	0.0006098	0.0002752	0.0005947
**Sy.x**	0.004295	0.005041	0.003386	0.004978

ND, non-determinable

Supplementary Table 5. Statistical analysis of the data presented in Fig. 4 by ANOVA

**Anova results**					
**Two-way RM ANOVA**	Matching: Stacked				
**Assume sphericity?**	No				
**Alpha**	0.05				
**Source of Variation**	% of total variation	P value	P value summary	Significant?	Geisser-Greenhouse's epsilon
**Time x agonist**	5.127	<0,0001	****	Yes	
**Time**	71.2	<0,0001	****	Yes	0.2536
**agonist**	14.35	<0,0001	****	Yes	
**Subject**	8.563	<0,0001	****	Yes	
**ANOVA table**	SS	DF	MS	F (DFn, DFd)	P value
**Time x agonist**	2162881	18	120160	F (18, 192) = 72,51	P<0,0001
**Time**	30036521	6	5006087	F (1,521, 48,68) = 3021	P<0,0001
**agonist**	6054030	3	2018010	F (3, 32) = 17,88	P<0,0001
**Subject**	3612152	32	112880	F (32, 192) = 68,12	P<0,0001
**Residual**	318174	192	1657		
**Data summary**					
**Number of columns (agonist)**	4				
**Number of rows (Time)**	7				
**Number of subjects (Subject)**	36				

**Number of missing values**	0				
**Multiple comparisons**					
**Number of families**	7				
**Number of comparisons per family**	6				
**Alpha**	0.05				
**Tukey's multiple comparisons test**	Mean Diff,	95,00% CI of diff,	Below threshold?	Summary	Adjusted P Value
**Row 1**					
**HHC R vs. HHC S**	23.95	-118,4 to 166,3	No	ns	0.9619
**HHC R vs. THC**	66.53	-49,63 to 182,7	No	ns	0.3789
**HHC R vs. WIN**	98.79	-12,22 to 209,8	No	ns	0.0878
**HHC S vs. THC**	42.58	-85,73 to 170,9	No	ns	0.768
**HHC S vs. WIN**	74.84	-49,31 to 199,0	No	ns	0.3226
**THC vs. WIN**	32.26	-52,93 to 117,5	No	ns	0.7013
**Row 2**					
**HHC R vs. HHC S**	94.79	-58,51 to 248,1	No	ns	0.321
**HHC R vs. THC**	141.2	1,899 to 280,4	Yes	*	0.0466

**HHC R vs. WIN**	-65.93	-203,0 to 71,17	No	ns	0.505
**HHC S vs. THC**	46.38	-72,86 to 165,6	No	ns	0.6782
**HHC S vs. WIN**	-160.7	-277,0 to -44,47	Yes	**	0.0064
**THC vs. WIN**	-207.1	-295,8 to -118,4	Yes	****	<0,0001
**Row 3**					
**HHC R vs. HHC S**	131.7	-53,52 to 317,0	No	ns	0.2159
**HHC R vs. THC**	203.9	35,64 to 372,1	Yes	*	0.0161
**HHC R vs. WIN**	-215.3	-387,5 to -43,04	Yes	*	0.0128
**HHC S vs. THC**	72.16	-77,46 to 221,8	No	ns	0.5212
**HHC S vs. WIN**	-347	-501,6 to -192,4	Yes	****	<0,0001
**THC vs. WIN**	-419.2	-547,9 to -290,4	Yes	****	<0,0001
**Row 4**					
**HHC R vs. HHC S**	135.5	-64,82 to 335,8	No	ns	0.2528
**HHC R vs. THC**	241.6	63,21 to 420,1	Yes	**	0.0072
**HHC R vs. WIN**	-269.6	-467,9 to -71,25	Yes	**	0.0065
**HHC S vs. THC**	106.1	-64,86 to 277,1	No	ns	0.3141
**HHC S vs. WIN**	-405.1	-597,4 to -212,8	Yes	****	<0,0001
**THC vs. WIN**	-511.2	-679,5 to -342,9	Yes	****	<0,0001

**Row 5**					
**HHC R vs. HHC S**	113.4	-93,51 to 320,4	No	ns	0.423
**HHC R vs. THC**	242.8	63,43 to 422,1	Yes	**	0.0071
**HHC R vs. WIN**	-316.4	-516,1 to -116,7	Yes	**	0.0017
**HHC S vs. THC**	129.3	-52,46 to 311,1	No	ns	0.2116
**HHC S vs. WIN**	-429.8	-631,6 to -228,1	Yes	****	<0,0001
**THC vs. WIN**	-559.2	-731,6 to -386,7	Yes	****	<0,0001
**Row 6**					
**HHC R vs. HHC S**	44.44	-203,6 to 292,5	No	ns	0.9548
**HHC R vs. THC**	233.6	26,44 to 440,7	Yes	*	0.0252
**HHC R vs. WIN**	-376.5	-612,2 to -140,8	Yes	**	0.0016
**HHC S vs. THC**	189.1	-25,76 to 404,0	No	ns	0.0928
**HHC S vs. WIN**	-420.9	-662,7 to -179,1	Yes	***	0.0007
**THC vs. WIN**	-610.1	-808,3 to -411,8	Yes	****	<0,0001
**Row 7**					
**HHC R vs. HHC S**	-78.04	-319,9 to 163,9	No	ns	0.7901
**HHC R vs. THC**	232.9	42,50 to 423,3	Yes	*	0.0144
**HHC R vs. WIN**	-481.6	-702,0 to -261,3	Yes	****	<0,0001
**HHC S vs. THC**	311	83,12 to 538,8	Yes	**	0.007

**HHC S vs. WIN**	-403.6	-653,8 to -153,3	Yes	**	0.0015
**THC vs. WIN**	-714.5	-917,7 to -511,4	Yes	****	<0,0001

**Test details**	Mean 1	Mean 2	Mean Diff,	SE of diff,	N1	N2	q	DF
**Row 1**								
**HHC R vs. HHC S**	2021	1997	23.95	49.67	9	9	0.682	15.74
**HHC R vs. THC**	2021	1954	66.53	40.09	9	9	2.347	14.37
**HHC R vs. WIN**	2021	1922	98.79	37.68	9	9	3.708	12.66
**HHC S vs. THC**	1997	1954	42.58	43.87	9	9	1.373	13.34
**HHC S vs. WIN**	1997	1922	74.84	41.68	9	9	2.54	11.74
**THC vs. WIN**	1954	1922	32.26	29.63	9	9	1.54	15.3
**Row 2**								
**HHC R vs. HHC S**	2183	2088	94.79	53.36	9	9	2.512	15.43
**HHC R vs. THC**	2183	2042	141.2	47.24	9	9	4.226	12.59
**HHC R vs. WIN**	2183	2249	-65.93	46.04	9	9	2.025	11.76
**HHC S vs. THC**	2088	2042	46.38	41.07	9	9	1.597	14.11
**HHC S vs. WIN**	2088	2249	-160.7	39.68	9	9	5.728	13.18
**THC vs. WIN**	2042	2249	-207.1	30.96	9	9	9.461	15.79
**Row 3**								

**HHC R vs. HHC S**	2410	2279	131.7	64.58	9	9	2.884	15.64
**HHC R vs. THC**	2410	2207	203.9	57.48	9	9	5.016	13.27
**HHC R vs. WIN**	2410	2626	-215.3	59.33	9	9	5.132	14.12
**HHC S vs. THC**	2279	2207	72.16	51.69	9	9	1.974	14.48
**HHC S vs. WIN**	2279	2626	-347	53.74	9	9	9.132	15.23
**THC vs. WIN**	2207	2626	-419.2	44.95	9	9	13.19	15.82
**Row 4**								
**HHC R vs. HHC S**	2573	2438	135.5	69.99	9	9	2.738	15.95
**HHC R vs. THC**	2573	2332	241.6	61.41	9	9	5.565	14.04
**HHC R vs. WIN**	2573	2843	-269.6	69.28	9	9	5.503	15.9
**HHC S vs. THC**	2438	2332	106.1	59.08	9	9	2.54	14.49
**HHC S vs. WIN**	2438	2843	-405.1	67.22	9	9	8.523	15.99
**THC vs. WIN**	2332	2843	-511.2	58.23	9	9	12.42	14.65
**Row 5**								
**HHC R vs. HHC S**	2667	2553	113.4	72.33	9	9	2.218	15.99
**HHC R vs. THC**	2667	2424	242.8	61.88	9	9	5.548	14.33
**HHC R vs. WIN**	2667	2983	-316.4	69.79	9	9	6.412	15.95
**HHC S vs. THC**	2553	2424	129.3	62.65	9	9	2.919	14.19

**HHC S vs. WIN**	2553	2983	-429.8	70.47	9	9	8.626	15.92
**THC vs. WIN**	2424	2983	-559.2	59.7	9	9	13.24	14.74
**Row 6**								
**HHC R vs. HHC S**	2785	2740	44.44	86.67	9	9	0.7252	15.97
**HHC R vs. THC**	2785	2551	233.6	70.98	9	9	4.654	13.58
**HHC R vs. WIN**	2785	3161	-376.5	82.35	9	9	6.465	15.95
**HHC S vs. THC**	2740	2551	189.1	73.37	9	9	3.645	13.21
**HHC S vs. WIN**	2740	3161	-420.9	84.42	9	9	7.051	15.83
**THC vs. WIN**	2551	3161	-610.1	68.22	9	9	12.65	14.03
**Row 7**								
**HHC R vs. HHC S**	2958	3036	-78.04	84.02	9	9	1.314	15.13
**HHC R vs. THC**	2958	2725	232.9	66.19	9	9	4.977	15.23
**HHC R vs. WIN**	2958	3440	-481.6	76.94	9	9	8.852	15.86
**HHC S vs. THC**	3036	2725	311	77.94	9	9	5.642	13.4
**HHC S vs. WIN**	3036	3440	-403.6	87.26	9	9	6.541	15.65
**THC vs. WIN**	2725	3440	-714.5	70.26	9	9	14.38	14.58

Supplementary Table 6. Statistical analysis of the data presented in Fig. 5 by ANOVA

Anova results

**Two-way RM ANOVA**	Matching: Stacked
**Assume sphericity?**	No
**Alpha**	0.05

**Source of Variation**	% of total variation	P value	P value summary	Significant?	Geisser-Greenhouse's epsilon
**Time x agonist**	4.528	0.0002	***	Yes	
**Time**	6.802	<0,0001	****	Yes	0.7975
**agonist**	5.781	0.4345	ns	No	
**Subject**	65.85	<0,0001	****	Yes	

**ANOVA table**	SS	DF	MS	F (DFn, DFd)	P value
**Time x agonist**	156142	18	8675	F (18, 192) = 2,835	P=0,0002
**Time**	234564	6	39094	F (4,785, 153,1) = 12,78	P<0,0001
**agonist**	199339	3	66446	F (3, 32) = 0,9364	P=0,4345
**Subject**	2270733	32	70960	F (32, 192) = 23,19	P<0,0001
**Residual**	587435	192	3060		

Data summary

**Number of columns (agonist)**	4
**Number of rows (Time)**	7
**Number of subjects (Subject)**	36

**Number of missing values**	0				
**Multiple comparisons**					
**Number of families**	7				
**Number of comparisons per family**	6				
**Alpha**	0.05				
**Tukey's multiple comparisons test**	Mean Diff,	95,00% CI of diff,	Below threshold ?	Summary	Adjusted P Value
**Row 1**					
**R-HHC vs. S-HHC**	38.63	-126,7 to 203,9	No	ns	0.9074
**R-HHC vs. THC**	8.796	-144,0 to 161,6	No	ns	0.9983
**R-HHC vs. WIN**	11.8	-132,9 to 156,5	No	ns	0.9951
**S-HHC vs. THC**	-29.83	-181,2 to 121,6	No	ns	0.9407
**S-HHC vs. WIN**	-26.82	-170,0 to 116,4	No	ns	0.9469
**THC vs. WIN**	3.008	-123,1 to 129,1	No	ns	0.9999
**Row 2**					
**R-HHC vs. S-HHC**	-11.08	-122,7 to 100,6	No	ns	0.9916
**R-HHC vs. THC**	-20.63	-157,9 to 116,7	No	ns	0.9707

**R-HHC vs. WIN**	-52.16	-209,8 to 105,4	No	ns	0.7636
**S-HHC vs. THC**	-9.551	-152,4 to 133,3	No	ns	0.9973
**S-HHC vs. WIN**	-41.09	-203,0 to 120,8	No	ns	0.8789
**THC vs. WIN**	-31.53	-207,8 to 144,7	No	ns	0.9547
**Row 3**					
**R-HHC vs. S-HHC**	17.42	-111,2 to 146,0	No	ns	0.9795
**R-HHC vs. THC**	-11.64	-164,8 to 141,5	No	ns	0.9961
**R-HHC vs. WIN**	-177.2	-360,6 to 6,082	No	ns	0.0595
**S-HHC vs. THC**	-29.06	-177,4 to 119,2	No	ns	0.9404
**S-HHC vs. WIN**	-194.7	-374,6 to -14,68	Yes	*	0.0326
**THC vs. WIN**	-165.6	-359,9 to 28,65	No	ns	0.1086
**Row 4**					
**R-HHC vs. S-HHC**	35.11	-86,56 to 156,8	No	ns	0.8398
**R-HHC vs. THC**	8.921	-111,2 to 129,1	No	ns	0.9964
**R-HHC vs. WIN**	-79.09	-247,3 to 89,17	No	ns	0.5401
**S-HHC vs. THC**	-26.19	-132,6 to 80,23	No	ns	0.894
**S-HHC vs. WIN**	-114.2	-275,8 to 47,38	No	ns	0.2106
**THC vs. WIN**	-88.01	-248,7 to 72,71	No	ns	0.4036

**Row 5**					
**R-HHC vs. S-HHC**	-8.598	-166,3 to 149,1	No	ns	0.9985
**R-HHC vs. THC**	4.088	-133,0 to 141,1	No	ns	0.9998
**R-HHC vs. WIN**	-64.75	-226,0 to 96,55	No	ns	0.6577
**S-HHC vs. THC**	12.69	-151,2 to 176,6	No	ns	0.9959
**S-HHC vs. WIN**	-56.15	-238,4 to 126,1	No	ns	0.8142
**THC vs. WIN**	-68.84	-236,1 to 98,40	No	ns	0.6449
**Row 6**					
**R-HHC vs. S-HHC**	20.84	-117,5 to 159,2	No	ns	0.9723
**R-HHC vs. THC**	-17.95	-226,7 to 190,8	No	ns	0.9939
**R-HHC vs. WIN**	-17.85	-164,9 to 129,2	No	ns	0.985
**S-HHC vs. THC**	-38.78	-248,0 to 170,4	No	ns	0.9457
**S-HHC vs. WIN**	-38.69	-186,8 to 109,4	No	ns	0.8759
**THC vs. WIN**	0.09302	-213,2 to 213,4	No	ns	>0,9999
**Row 7**					
**R-HHC vs. S-HHC**	14.28	-87,46 to 116,0	No	ns	0.977
**R-HHC vs. THC**	-4.98	-114,5 to 104,5	No	ns	0.9992
**R-HHC vs. WIN**	-35.03	-184,1 to 114,1	No	ns	0.9008
**S-HHC vs. THC**	-19.26	-119,5 to 80,93	No	ns	0.9447

**S-HHC vs. WIN**	-49.3	-193,6 to 94,98	No	ns	0.7446			
**THC vs. WIN**	-30.05	-178,4 to 118,3	No	ns	0.9327			
**Test details**	Mean 1	Mean 2	Mean Diff,	SE of diff,	N1	N2	q	DF
**Row 1**								
**R-HHC vs. S-HHC**	605	566.4	38.63	57.77	9	9	0.9456	16
**R-HHC vs. THC**	605	596.2	8.796	53.17	9	9	0.2339	15.4
**R-HHC vs. WIN**	605	593.2	11.8	49.86	9	9	0.3348	14.15
**S-HHC vs. THC**	566.4	596.2	-29.83	52.73	9	9	0.8002	15.48
**S-HHC vs. WIN**	566.4	593.2	-26.82	49.39	9	9	0.7681	14.26
**THC vs. WIN**	596.2	593.2	3.008	43.92	9	9	0.09687	15.51
**Row 2**								
**R-HHC vs. S-HHC**	569.7	580.8	-11.08	38.92	9	9	0.4024	15.62
**R-HHC vs. THC**	569.7	590.3	-20.63	47.05	9	9	0.62	13.58
**R-HHC vs. WIN**	569.7	621.8	-52.16	53.27	9	9	1.385	12.29
**S-HHC vs. THC**	580.8	590.3	-9.551	49.5	9	9	0.2729	14.8
**S-HHC vs. WIN**	580.8	621.8	-41.09	55.45	9	9	1.048	13.5
**THC vs. WIN**	590.3	621.8	-31.53	61.42	9	9	0.726	15.57

**Row 3**								
**R-HHC vs. S-HHC**	594.8	577.4	17.42	44.9	9	9	0.5487	15.84
**R-HHC vs. THC**	594.8	606.5	-11.64	53.24	9	9	0.3092	15.28
**R-HHC vs. WIN**	594.8	772.1	-177.2	62.74	9	9	3.995	13.45
**S-HHC vs. THC**	577.4	606.5	-29.06	51.28	9	9	0.8014	14.59
**S-HHC vs. WIN**	577.4	772.1	-194.7	61.09	9	9	4.506	12.65
**THC vs. WIN**	606.5	772.1	-165.6	67.46	9	9	3.472	15.11
**Row 4**								
**R-HHC vs. S-HHC**	657.4	622.3	35.11	42.34	9	9	1.173	15.38
**R-HHC vs. THC**	657.4	648.5	8.921	41.74	9	9	0.3022	15.16
**R-HHC vs. WIN**	657.4	736.5	-79.09	57.98	9	9	1.929	14.17
**S-HHC vs. THC**	622.3	648.5	-26.19	37.19	9	9	0.9957	15.98
**S-HHC vs. WIN**	622.3	736.5	-114.2	54.79	9	9	2.948	12.57
**THC vs. WIN**	648.5	736.5	-88.01	54.33	9	9	2.291	12.3
**Row 5**								
**R-HHC vs. S-HHC**	643.1	651.7	-8.598	54.48	9	9	0.2232	14.48
**R-HHC vs. THC**	643.1	639	4.088	47.82	9	9	0.1209	15.76
**R-HHC vs. WIN**	643.1	707.9	-64.75	55.61	9	9	1.647	14.24
**S-HHC vs. THC**	651.7	639	12.69	57	9	9	0.3147	15.33

**S-HHC vs. WIN**	651.7	707.9	-56.15	63.68	9	9	1.247	15.98
**THC vs. WIN**	639	707.9	-68.84	58.09	9	9	1.676	15.14
**Row 6**								
**R-HHC vs. S-HHC**	647.8	627	20.84	48.35	9	9	0.6094	16
**R-HHC vs. THC**	647.8	665.7	-17.95	70.64	9	9	0.3593	12.4
**R-HHC vs. WIN**	647.8	665.6	-17.85	51.32	9	9	0.492	15.75
**S-HHC vs. THC**	627	665.7	-38.78	70.91	9	9	0.7735	12.52
**S-HHC vs. WIN**	627	665.6	-38.69	51.69	9	9	1.059	15.81
**THC vs. WIN**	665.7	665.6	0.09302	72.96	9	9	0.001803	13.38
**Row 7**								
**R-HHC vs. S-HHC**	598.4	584.1	14.28	35.41	9	9	0.5702	15.41
**R-HHC vs. THC**	598.4	603.4	-4.98	38.27	9	9	0.184	15.99
**R-HHC vs. WIN**	598.4	633.4	-35.03	51.09	9	9	0.9695	13.55
**S-HHC vs. THC**	584.1	603.4	-19.26	34.9	9	9	0.7803	15.54
**S-HHC vs. WIN**	584.1	633.4	-49.3	48.62	9	9	1.434	12.04
**THC vs. WIN**	603.4	633.4	-30.05	50.74	9	9	0.8374	13.36
